# The Global Socioeconomic Burden of Obstructive Sleep Apnea: A Comprehensive Review

**DOI:** 10.3390/healthcare13172115

**Published:** 2025-08-26

**Authors:** Paolo Zappalà, Mario Lentini, Salvatore Ronsivalle, Salvatore Lavalle, Luigi La Via, Antonino Maniaci

**Affiliations:** 1 Department of Medicine and Surgery, Kore University of Enna, 94100 Enna, Italy; paolo.zappala@unikore.it (P.Z.); mario.lentini@unikore.it (M.L.); salvatore.ronsivalle@aspct.it (S.R.); salvatore.lavalle@unikore.it (S.L.); 2 UOC Otolaryngology, Asp Ragusa, 97100 Ragusa, Italy; 3 UOC Otolaryngology, “Gravina e Santo Pietro” Hospital, ASP Catania, 95041 Caltagirone, Italy; 4 UOC Otolaryngology, “Umberto I” Hospital, ASP Enna, 94100 Enna, Italy; 5 Department of Anesthesia and Intensive Care 1, University Hospital Policlinico “G. Rodolico-San Marco”, 95123 Catania, Italy; luigilavia7@gmail.com

**Keywords:** obstructive sleep apnea, healthcare economics, global health burden, treatment accessibility, telemedicine, healthcare disparities, cost-effectiveness analysis

## Abstract

**Relevance**: Obstructive sleep apnea (OSA) is a major public health problem with significant social and economic consequences. With increasing prevalence associated with urbanization and aging, untreated OSA is a considerable burden to the healthcare system, work productivity, and accident costs. **Objectives**: To analyze the global socioeconomic burden of OSA and evaluate epidemiological, economic, and healthcare policy perspectives across different regions and income levels. **Materials and Methods**: We conducted a narrative comprehensive review of published studies and WHO reports, covering direct medical costs, indirect social costs, and the cost-effectiveness of both existing and emerging diagnostic and therapeutic techniques. **Results**: OSA is estimated to afflict some 936 million adults around the world, and if it remains untreated, OSA results in 2.5 times higher healthcare costs compared to non-OSA individuals. The annual societal cost of untreated OSA in the U.S. now exceeds USD 150 billion, when considering direct medical expenses, productivity losses, and accident-related costs. Recent studies also highlight significant global costs, with annual per-patient estimates up to EUR 28,000 in the U.S. and EUR 1700–5000 in Europe. The inequality of treatment access continues between the affluent and the poor regions. Novel approaches as AI diagnostics and telemedicine, hold promise for reducing costs and improving treatment adherence among underserved populations with limited access to conventional care. **Discussion**: This review underscores the importance of uniform care throughout the world, timely diagnosis initiatives using portable technology, and scalable technological solutions to help reduce the social toll of OSA. Policymaker support, public education campaigns, and insurance changes are necessary to optimize both the cost and effectiveness of OSA management worldwide.

## 1. Introduction

Obstructive sleep apnea (OSA) is a common sleep disorder with substantial socioeconomic implications worldwide [1,2,3]. Obstructive sleep apnea syndrome (OSAS) is a chronic respiratory condition characterized by complete or partial obstruction of the upper airway during sleep [1], and it is a major public health issue, affecting approximately 936 million people worldwide, aged 30–69 years [2,3,4,5]. OSA adversely affects individual health and imposes a significant burden on healthcare systems and economies through increased healthcare utilization, reduced productivity, and accident-related costs [3,6,7]. The prevalence of OSA is rising globally, as shown in recent epidemiological studies [7,8,9]. The global prevalence rose by 12% compared to that of the previous decade, especially in the rapidly urbanizing areas of Asia and Latin America [2].

Although OSA is estimated to affect 936 million adults worldwide, it is important to acknowledge that the majority of our knowledge is based on findings derived from studies in high-income countries. The epidemiology, healthcare access and treatment options, and economic consequences are also likely to significantly vary depending on local socioeconomic considerations, especially in LMICs where diagnostic and treatment resources are generally lacking. Several factors have been implicated in this trend, including the worldwide obesity epidemic, aging populations worldwide, and increased diagnostic capacity [3]. OSA is a multifactorial disorder that comprises multiple anatomic and neuromuscular components. During sleep, the airway is prone to collapse because of its poor tonicity in the neuromuscular structures of the upper airway and selected anatomy. These occurrences result in intermittent hypoxemia, sleep fragmentation, and stimulation of the sympathetic nervous system, activating a cascade of physiological processes [4]. New pathways in which OSA induces systemic inflammation and oxidative stress as a basis for many comorbid diseases have been recently recognized [5]. There are major variations in the OSA risk factors between populations, but obesity remains the most common modifiable risk factor. A cross-cultural study in 28 countries found that in Western populations, obesity accounts for ≈60% of OSA cases, whereas in Asian populations, craniofacial factors contribute risk to a greater extent [6]. Age, male sex, and family history are well-established non-modifiable risk factors, but women have an increased susceptibility to RA post-menopausally [7]. The socioeconomic effects of untreated OSA are profound and complex [8]. The financial consequences of untreated OSA have been updated in recent studies, with total annual societal costs in the U.S. now exceeding USD 150 billion [8,9]. This includes ~USD 95 billion in direct medical costs and additional indirect losses from workplace productivity, accidents, and comorbid conditions [10,11]. More recent data from low- and middle-income countries suggest that the economic burden of a pandemic may be greater in low-income settings where diagnostic and treatment services are often scarce [9]. The economic impact is further increased by OSA and the associated comorbidities (such as CVD, type 2 DM, and depression). According to a systematic review published in 2024, untreated moderate to severe OSA patients incur 2.5 times the healthcare costs of those without OSA [10]. The repercussions extend beyond healthcare systems to work productivity and safety. Scientific evidence indicates that untreated OSA is associated with an up to 2.5-fold increase in workplace accidents and a 77% decrease in productivity [11]. Although the disease burden is significant, OSA is highly underdiagnosed worldwide, with estimated rates of under-identification exceeding 80% in some populations [12].

Moreover, recent cost-of-illness studies underscore the escalating economic burden of untreated OSA globally. In Italy alone, annual societal costs attributable to OSA and its comorbidities range from EUR 10.7 to EUR 32.0 billion [11]. In Australia, OSA accounted for ~USD 13.1 billion in annual costs (2019–2020), highlighting the worldwide scale of the problem. This diagnostic and treatment delay is a major public health challenge, particularly in resource-limited settings. New trends in technology for diagnostic devices and treatment modalities may offer some hope for better access to care, but cost-related issues are an insurmountable obstacle for them to be implemented more widely [13]. A global perspective on the socioeconomic impact of OSA reveals massive differences in the burden of disease and care deferred, and greatly unequal opportunities. As lower- and middle-income countries elsewhere labor to hold the line on the cost of delivering healthcare and matching it with treatment for populations, their counterparts in the world struggle with more basic problems of sexual health: sufficient diagnosis and access to treatments. These explorations of context-specific disparities may be important in fostering scalable interventions and policy recommendations to address the global burden of OSA [14]. Previous economic burden of OSA reviews have been limited in scope to specific geographic areas (predominantly North America and Western Europe) or elements of economic impact (direct healthcare expenditures or productivity reduction). Despite their contribution, these studies tend to suffer from several important limitations: (1) they rarely offer a truly global view taken between high income settings and low resource settings; (2) they typically do not take on board the rapidly shifting technological backdrop that is reshaping the landscape of OSA diagnosis and treatment; (3) they rarely account for the essential interrelationship between socioeconomic features, configurations of healthcare systems, and the availability of treatment; and (4) they generally do not provide actionable policy-specific advice founded on economic analysis.

This review aimed to summarize the entire published data on the socioeconomic burden of OSA, considering a global perspective, and the differences observed in social and healthcare impact according to continent. By examining the evidence and ongoing changes at our disposal, we aimed to evaluate socioeconomic and patient outcomes in different practice environments.

## 2. Materials and Methods

This manuscript follows a structured narrative review methodology designed to comprehensively investigate the worldwide social and economic impact of OSA, focusing on costs, health burden, and access to care. While maintaining the flexibility of a narrative approach, we implemented a rigorous and transparent process for literature identification, selection, and synthesis.

### 2.1. Search Strategy

A comprehensive search strategy was developed in consultation with a health sciences librarian. We systematically searched three electronic databases: PubMed, Scopus, and Web of Science for studies published from January 2010 to December 2024. This timeframe was selected to capture contemporary economic analyses while including seminal works that established fundamental concepts in OSA socioeconomics.

The following search terms and Boolean operators were used:

- Primary terms: “obstructive sleep apnea” OR “OSA” OR “sleep-disordered breathing”.
- Combined with AND (“economic burden” OR “healthcare costs” OR “treatment access” OR “cost-effectiveness” OR “global health” OR “socioeconomic” OR “productivity loss” OR “absenteeism” OR “presenteeism” OR “quality of life” OR “healthcare utilization” OR “insurance coverage”).

### 2.2. Eligibility Criteria

Eligible studies were defined according to the following inclusion criteria:

- Original peer-reviewed research, review, or meta-analysis;
- Articles written in English;
- The article focuses on the economic burden, treatment, healthcare policy, or access to care associated with OSA.
- Official reports from major health organizations containing relevant data on OSA epidemiology, economic burden, or healthcare policy

Exclusion criteria were:

- Opinions and commentaries, letters, and case reports;
- Non-English publications;
- Studies with OSA not being the main focus in the study, e.g., studies that did not specifically address economic or health system issues.

### 2.3. Study Selection Process

The study selection process followed a two-stage approach:

Initial screening: Two investigators (P.Z. and M.L.) independently screened titles and abstracts of all retrieved records against the eligibility criteria. Disagreements were resolved through discussion with a third reviewer (A.M.).

Full-text review: The same two investigators independently reviewed full texts of potentially eligible articles. Reasons for exclusion at this stage were documented (common reasons included: lack of economic data, focus on clinical outcomes only, or insufficient methodological detail).

**Figure 1:**
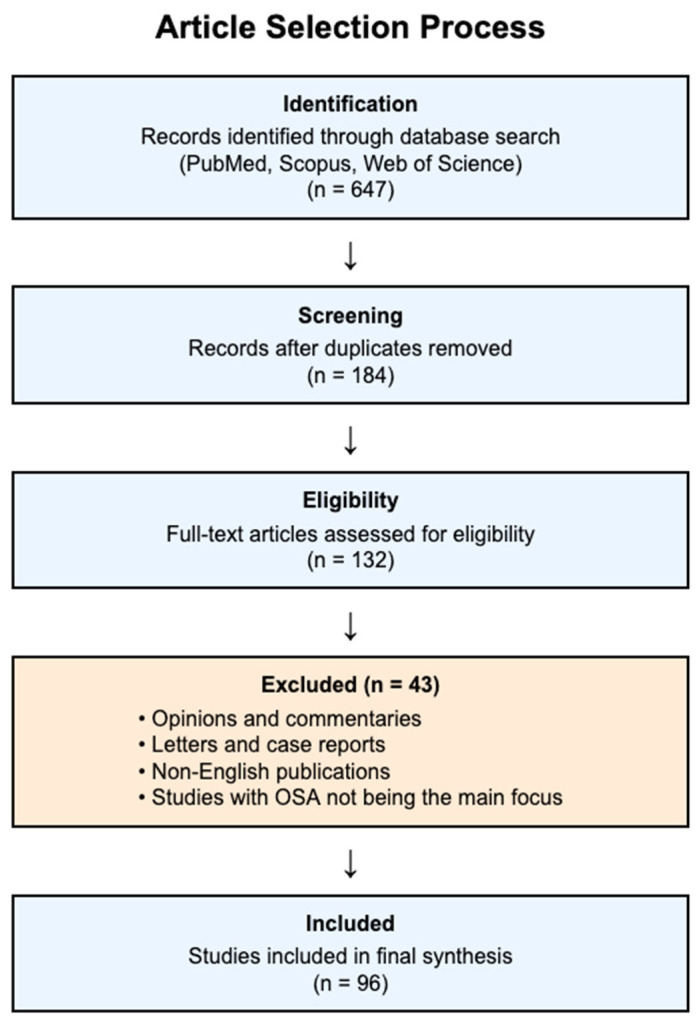
Study selection process.

After relevance screening, duplicate removal, and title/abstract screening, there were initially about 647 articles found (Figure 1).

Full-text articles for 132 manuscripts were reviewed, and 96 were retained for the final synthesis after screening of titles and abstracts.

### 2.4. Quality Assessment and Context of Evidence

One of the main limitations in the current literature on the economic burden of OSA is the heterogeneity of the methods used, economic contexts, and reporting issues. To overcome this, we used an organized process of critical appraisal in our analysis. For each item concerning cost or economic analysis, we recorded the original currency, year of study, discount factor used, and viewpoint (patient, payer, or societal) of the analysis. All the estimates were inflation-adjusted to 2024 US dollars using country-specific healthcare inflation indices and purchasing power parity conversion factors derived from the World Bank to make their comparisons more relevant when we cited costs in more than one country or at more than one time period. For cost-effectiveness analyses, we assessed the assumptions, time horizon, comparators, and willingness-to-pay thresholding, recognizing that these are highly dependent on the healthcare setting and can lead to very different values. We examined whether research took into account direct and indirectly related costs (lost productivity, accidents) or included all the costs of OSA, in view of the fact that a partial economic analysis usually underestimates the overall societal burden of the disease. Throughout our synthesis, we explicitly acknowledge when evidence quality is limited for specific regions or populations, particularly noting the scarcity of robust economic data from low- and middle-income countries. Rather than presenting findings as definitive, we frame conclusions according to the strength of supporting evidence, highlight methodological limitations of source studies, and identify critical knowledge gaps that should inform future research priorities.

## 3. The Economic Burden of OSA: Direct and Indirect Costs

OSA represents an economic burden due to direct healthcare costs and important indirect costs to society. Information on the processes and costs of some of these common diseases and conditions indicates that the direct and indirect costs of those conditions have a direct impact on the larger burden on health/social-care systems.

**Figure 2:**
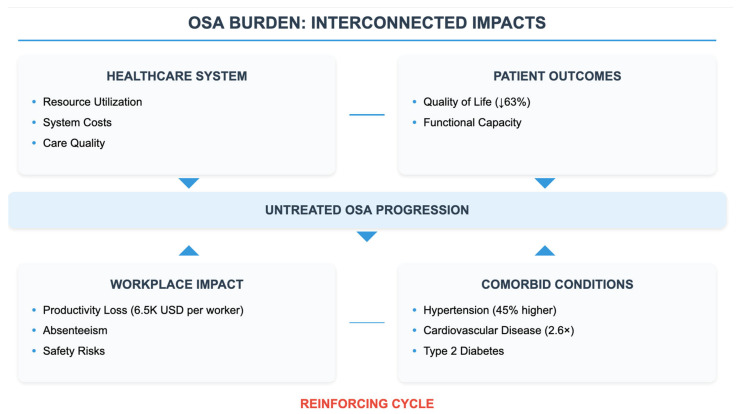
Economic impact of OSA and interconnected influence on working productivity.

In light of the amount of good news on the economy of OSA, direct comparison of the surveys is complicated by differences in methodology. The majority of economic burden studies have been conducted in high-income countries with established healthcare systems, leaving critical information voids in the costs of OSA in low-resource settings. The economic numbers presented have to be interpreted with caution for their application to LMICs, where the healthcare infrastructure, payment system, and other competing health needs make for a significantly different economic environment. Over the last decade, direct healthcare costs attributable to the diagnosis and treatment of OSA have steadily increased. When costs in several healthcare systems were analyzed, polysomnographic and home diagnostic testing amounted to 23% of the initial costs of healthcare for these patients [15]. The average costs of a diagnosis are from USD 600 to USD 6000 per diagnostic procedure and healthcare location [16]. As new diagnostics are developed, including at-home testing technologies using AI, early pilot studies suggest these might reduce diagnostic costs, though the magnitude of savings (estimated at up to 40% in small studies) requires validation in larger, real-world implementations [17]. The cost of treatment, or CPAP therapy cost, bears strongly on the direction of direct healthcare spending. In recent analyses of the market, the annual costs for CPAP equipment (purchase or rent, supplies, and maintenance) varied from USD 1000 to USD 3500 [8]. The accessibility of other treatments (e.g., mandibular advancement devices (MADs) and surgeries) advances now with additional costs (according to the technique and location, procedural costs: USD 10,000 to USD 40,000) [18] (Figure 2).

Despite the impressive economic data on OSA, direct comparisons between studies are difficult due to methodological disparities. Indeed, many cost studies do not strictly control for confounding variables, especially intricate interactions between OSA and comorbid systems, which may overestimate OSA-specific costs. Furthermore, most of the existing economic burden studies have been performed in high-income countries with established healthcare systems, resulting in important knowledge gaps on the actual costs of HSV in resource-poor settings with large variations in accessibility to diagnosis and treatment.

OSA is highly associated with various comorbid conditions, which results in a significant increase in healthcare costs. In a large observational analysis of healthcare claims, the estimated out-of-pocket cost of OSA patients was approximately 2.3 times higher for untreated patients versus treated patients (because of comorbidities of the cardiovascular system) [19]. In the United States, OSA-associated comorbidity costs are estimated to contribute significantly to total healthcare spending. Untreated OSA patients incur ~2.5× higher annual healthcare costs compared to those without OSA [8,15,20]. Additionally, direct medical costs of sleep disorders, largely OSA, were ~USD 95 billion/year as of 2018 [9]. Several comorbid conditions with economic consequences are also highlighted in recent studies. Healthcare utilization every year is remarkably greater for these patients with OSA + and type 2 diabetes (23% greater than diabetes alone) [20]. Patients with OSA and concomitant cardiovascular disease exhibit a 34% rise in healthcare utilization and costs [21]. These indirect costs—the financial strain caused by lost workplace productivity and absenteeism—have been determined as substantial. A multinational study across 15 countries reported that work output losses associated with OSA amount to USD 86.9 billion per annum in the US and EUR 50 billion across the EU [22]. About 70% of this lost productivity is associated with presenteeism—employees coming to work, but not fully functioning because of the negative effects of OSA-related fatigue [23]. Patients not treated for OSA average 13.3 days of sickness a year more than treated patients [24]. Workplace-based screening and therapy implementation have been linked with a reduction in absenteeism related to OSA of 40% [25]. Accidents associated with OSA generate a significant social–economic burden. Untreated OSA was associated with a 2.4-fold risk of MVA, and the costs related to crash injuries were estimated to be USD 16 billion per year I States [26]. OSA-related traffic accidents in the European Union are estimated at EUR 21 billion annually [27]. The financial and legal effects of job accidents involving OSA-induced fatigue are substantial. As per a series of recent occupational safety studies, the incidence of workplace accidents among workers with untreated OSA is 1.9 times in excess, which also accounts for this higher propensity for insurance and litigational costs for employers [28]. It is relevant to explore the association between OSA and work productivity. Current estimates of productivity loss could be overstated due to reporting and respondent bias, such that individuals with more severe symptoms are more likely to participate in the studies. Also, the subjective base (personal perception) of presenteeism measurements and the various methods for measuring productivity losses used in the different groups of workers contribute to great variability in the economic impact estimates.

Mandatory OSA screening programs for high-risk occupations (such as commercial drivers and heavy equipment operators) are estimated to result in potential cost savings of USD 4000–8000 per worker per year due to prevented accidents [29].

## 4. OSA Treatment Strategies: Cost-Effectiveness and Accessibility

CPAP therapy is now the recognized gold standard for OSA. In the United Kingdom, an ICER of USD 15,915 per QALY was reported for CPAP in adults with moderate or severe OSA based on an economic evaluation [30]. Studies in Europe and North America comparing mandibular advancement devices (MADs) have also established cost-effectiveness in the vicinity of USD 24,300/QALY for custom-made MADs [31,32]. The ICERs are derived from health economic models that only have applicability in high-income countries and not directly in low- and middle-income settings. The claimed cost-effectiveness of CPAP warrants critical review. Estimates of the ICERs for OSA interventions often use quality-of-life improvements based on small clinical trials with short follow-up times, which may overestimate long-term benefits. In addition, these economic models often employ ideal compliance rates, which are also higher than adherence trends in real life, particularly in low-resource settings where challenges of adherence to CPAP are most severe. Whether these cost-effectiveness ratios can be extrapolated to other healthcare systems is still in doubt, as they generally do not consider infrastructural constraints and culturally specific barriers to implementation.

Therefore, care needs to be taken in extrapolating these results to other healthcare systems.

**Figure 3:**
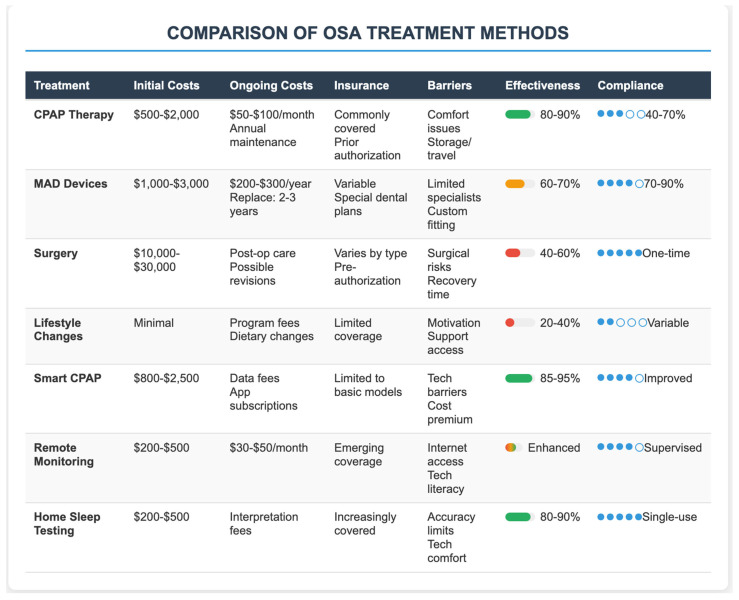
OSA treatment modalities: cost–benefit and implementation analysis.

It is suggested that contemporary MADs offer more appealing economic profiles over the long term, with the durability of the appliance and reduction in maintenance costs [33]. Surgical therapies, including UPPP and hypoglossal nerve stimulation, could have heterogeneous cost-effectiveness (Figure 3).

The patient selection bias is the most evaluated effect sought to explain the potential difference, and the results of all studies indicate that patients who are optimally selected are at least as cost-effective as those treated with CPAP [34]. The cost-effectiveness of new minimally invasive surgical methods with shorter recovery times and fewer complications is certainly promising [35]. Access to OSA management may vary according to healthcare systems. A multicountry study reported that cost remains a major barrier, and 42% of the patients interrupted therapy for economic reasons [36]. Compliance costs of CPAP therapy with insurance are estimated to range from 400 to 400–800 and 800 per year on average [37]. Delayed diagnosis and treatment, especially among poorer people, have been linked to high-deductible health plans. Our analysis of insurance claims data also demonstrated that among those with HDHPs, 30% were less likely to initiate CPAP therapy within the first six months of an initial diagnosis [38]. The duality of public and private health systems is another important factor in determining access to and quality of OSA treatment. The consult and treatment lag times are also, in general, longer in the public healthcare system, with a mean delay of at least 8 months to 1 year at some sites [39]. However, they provide broader protection after beginning therapy [40]. Treatment is almost always faster under the private health system, but more expensive. Across 15 countries, private systems had 60% shorter time from diagnosis to treatment compared with public systems, but with 2.5-fold higher out-of-pocket expenses [41]. In this review, we focus on novel technologies that have emerged in the past few years, opening new uncharted territories in cost-effective and costless OSA management. Cloud-enabled and remotely monitored CPAP devices are characterized by 23% less total cost due to lower in-office visits and better compliance monitoring [42]. The programs have met with success in rural and underserved regions [43]. AI-integrated auto-titrating devices have offered a more affordable alternative to conventional CPAP titration, decreasing capital costs by up to 40% [44]. While the machine-learning-based tools like these apps are very attractive for third-party and payer interest in the benefits of optimizing long-term compliance and decreasing the cost of ongoing management [45]. Smartphones and consumer sleep tracking devices became new screening tools that are more accessible for screening and monitoring [3,4]. Although they may have the potential to reduce diagnosis costs, studies indicate they need to be used to complement, rather than replace, standard diagnostic practices [46]. The cost–benefit of such human enhancements will vary from healthcare system to healthcare system. The new agent has been rapidly spread and introduced into high-income countries, while distribution and delivery of new agents to low- to middle-income countries, with limited infrastructure and financial resources, is slow [47]. However, the likelihood that the same technologies adapted for limited resources are less cost-effective has already been surpassed [48]. Although technological advances in the treatment of OSA are encouraging, the cost–benefit of these technologies is still questionable and should be examined. Several trials focusing on AI-augmented diagnostics and smart CPAP devices are industry-supported, thus increasing the risk of reporting biases. Second, the expectation that technology will simply ‘solve’ care disparities for low-resource contexts overlooks the ‘digital divide’ and can even serve to further stratify access to healthcare if technology rollout is not consciously developed to engage with the socioeconomic determinants of access.

## 5. Regional Differences in OSA Burden and Treatment Impact

Variation in healthcare systems, socioeconomic status, and population profiles, among other factors, results in wide variation in the prevalence and provision of treatment to OSA and cost across the world. The current paper sheds some light on this regional variation and its consequences from the management standpoint of OSA. The OSA prevalence in North America is one of the highest in the literature, where current estimates are that 34% of men and 17% of women have moderate-to-severe OSA [49]. The U.S. is the first adopter of world-class procedures and techniques for treatments; for example, by 2020, 78% of the average sleep centers owned world-class equipment like patient remote monitoring systems [50]. But relative to high-quality care, these gaps in access are enormous. Differences are also observed among various ethnic minorities, and among rural areas, where the diagnosed rate is on average 40% lower than in areas of urban residence [51]. Longer wait times yet even more inequity of access, yet better healthcare outcomes in human lives by some measures, 8–12 months to Specialist Sleep Services [52].

**Figure 4:**
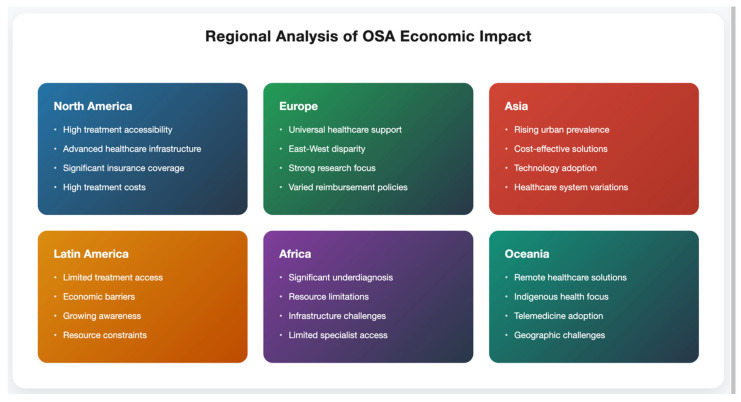
Regional disparities in OSA healthcare delivery and economic impact. Color-gradient visualization representing region-specific OSA management characteristics across six continental regions.

The overreliance on North American populations for research adds serious bias to our understanding of OSA’s global burden. The application of prevalence figures, economic burden estimates, and treatment effectiveness data from predominantly white, urban, insured US populations is questionable at best when applied to other parts of the world. This bias in research methodology, being Western-centric, leaves a gap in the literature for understanding the differences in phenotypic expression, economic, and best management strategies for OSA in different population groups. Public healthcare in Western Europe is well-established, and OSA therapy is covered to some extent or fully (70% to 100% of the cost) [53]. Treatment modality: There were marked differences among mainland countries in the form of diagnosing and treating SA, with consultation of the episcreen for diagnosis reaching a peak of 82% of cases in Northern European countries, but only 65% in Southern Europe, where laboratory studies were more popular [54]. Many Eastern European areas have much to do because the availability of many specialized centers is lacking, and the accessibility of treatment is poor. Recent assessments indicate that only 23% of patients diagnosed in Eastern Europe receive adequate treatment as opposed to 68% in Western Europe [55]. OSA has specific risk characteristics in Asian populations, such as lower BMI cut-offs for the development of disease and a higher percentage of positional OSA [56]. A rise in prevalence has also been observed in studies that report an annual increase of 23.4% of diagnosed cases in rapidly Westernizing populations in recent years [57] (Figure 4).

Japan and South Korea have the added advantage of being early adopters of low-cost and disruptive models that rely on simplified diagnostic algorithms and mHealth technology, lowering the cost of diagnosis by up to 45% [58]. However, few areas in Southeast Asia have poor access to services, e.g., with one polysomnographic laboratory for 1.2 million residents elsewhere [59]. For instance, there are important challenges in OSA management in several high out-of-pocket cost, low-specialized centers in Latin American countries. Based on a multicentric study spanning six countries, only 32% of eligible patients can be treated with CPAP [60]. Creative solutions spring up in the context of limited financial resources, in the form of shared CPAP programs and community screening projects [61]. OSA challenges in Africa. In the continent of Africa, the current state of OSA is marred by high rates of underdiagnosis and poor treatment. Fewer than 50 accredited stress [62] centers have been reported/estimated on the continent. Innovative approaches such as mobile diagnostic units and simplified screening tools have shown promise in low-resource contexts [63].

Besides the infrastructural barriers, management of OSA in LMIC, as in SSAs and some areas of LATAM, is significantly influenced by cultural, sociopolitical, and educational factors. In at least some, the awareness of sleep disordered breathing is low, with OSA symptoms ascribed to age, obesity, or other stigmatized syndromes, dampening demand for care. Snoring or drowsiness could still be viewed as a cultural characterization rather than being considered a medical issue; people want to avoid the social embarrassment associated with a sleep study or sleeping with a CPAP apparatus in a shared living space.

At a sociopolitical level, fragmented health governance, little priority has been given to sleep health in national policy, and failure to mainstream sleep medicine into primary healthcare are major factors for this underdiagnosis. Further, public health messaging may not penetrate rural populations or be presented in appropriate language and cultural contexts. Left unchecked, economic inequalities, poor health insurance systems, and corruption in procurement chains can further slow the distribution of affordable diagnostic tools and therapies. Tackling OSA effectively in these environments will require investment in infrastructure and community engagement, culturally sensitive education, and policy changes that raise the profile of sleep health on national health agendas.

A regional analysis conducted by Nyamande et al. estimated that productivity loss due to untreated OSA would be up to 2.2% of the GDP in specific sub-Saharan African nations. Although this figure indicates the potential economic impact, it must be considered with some caution and arguably cannot be extrapolated to the whole continent, given the lack of regional data and the difference in extrapolation techniques used in local or regional studies [64]. Public–private partnership is considered an option but remains confined to urban areas only [65].

A lack of generalizable data from sub-Saharan Africa is an important deficiency in global OSA research. A small handful of studies have been conducted and are often based only on small urban samples that are not representative of the diversity of regional populations. This lack of data results in a circular issue in which the economic burden goes unrecorded, thus preventing policy prioritization and restricting the availability of funds for research and clinical infrastructure. The extrapolation of estimates from Western countries could have led to a considerable overestimation of the underlying burden in the region and did not consider various healthcare-seeking behaviors, social and cultural attitudes towards sleep disorders, and competing health priorities.

Australia and New Zealand also have well-developed OSA services with an emphasis on indigenous population requirements [66]. Telemedicine provision has opened access to remote areas, because of which diagnostic delays decreased by 65% [67]. The area of OSA research in native populations is strong as an area, revealing crucial genetic and anatomical issues affecting treatment options [68]. The greatest success has been achieved with telemonitoring devices that have resulted in dramatically improved adherence rates (28% in rural trials) [69]. Cost-effectiveness studies suggest that the group-based treatment models could decrease the per-patient cost by 35% contrasts to traditional care in the remote areas of the country [70].

## 6. Policy Recommendations for Reducing the Socioeconomic Impact of OSA

The development of effective policies to address the socioeconomic burden of OSA requires a comprehensive, multi-stakeholder approach that considers both immediate and long-term impacts.

### 6.1. Strategies to Improve Early Diagnosis and Reduce Long-Term Healthcare Costs

**Figure 5:**
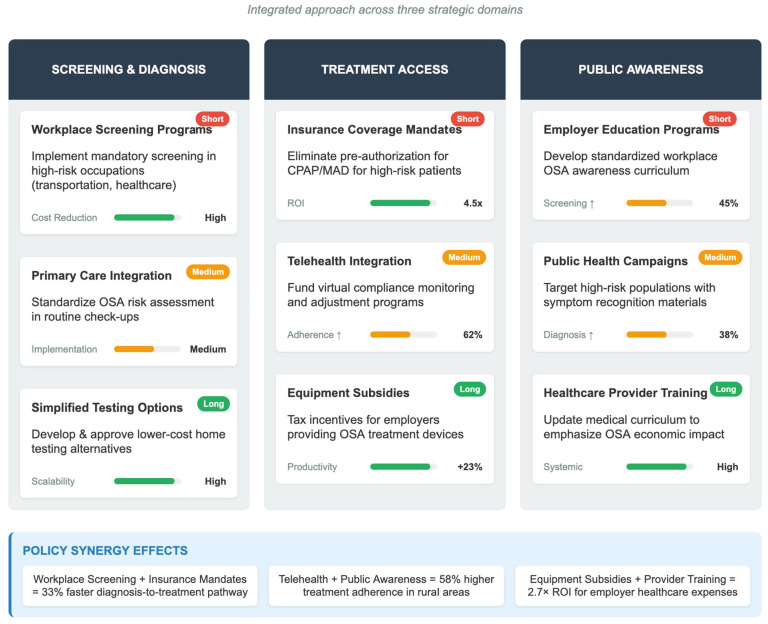
Policy recommendations for OSA and Economic impact reduction.

Early intervention methods have shown great promise in alleviating the long-term healthcare costs associated with OSA. Standardized screening protocols have been employed within primary care to reduce diagnostic delays and healthcare costs by up to 45% [71]. Machine learning algorithms used to develop risk stratification models have led to a 38% improvement in diagnostic efficiency and a reduction in unnecessary testing [72]. Cost-effectiveness analyses also favor the adoption of two-stage diagnostic algorithms that incorporate home sleep testing followed by selective laboratory-based polysomnography. This method has shown promising cost-savings of USD 2100/patient diagnosed with preserved diagnostic effectiveness [73] (Figure 5).

However, incorporating electronic health record-integrated screening tools may help identify high-risk patients, and one large healthcare system found that this approach increased early diagnosis rates by 52% [74].

The incorporation of OSA treatment into established chronic disease programs scores particularly well in terms of outcome and cost–benefit. The UK National Diabetes Prevention Programme’s pilot projects have included routine testing for OSA in their patients with suboptimal glycaemic control, with a subsequent 23% rise in OSA diagnoses coupled with lowering HbA1c levels upon commencement of treatment. Moreover, the U.S.-based Kaiser Permanente health system has integrated OSA screening into its cardiovascular disease program, resulting in OSA diagnosis and a 17% decrease in heart failure-related hospital readmissions. These cases illustrate how migrating OSA care into chronic disease care pathways is matched to patient risk profiles and can ensure the efficient use of healthcare resources through foreshortening of systems, coordinated care, and multidisciplinary interventions. Although such policy responses are promising for high-income contexts, their applicability in LMICs necessitates careful tailoring to local circumstances. Responses need to consider constrained health systems, varying modalities of health financing, workforce capacity limitations, and cultural issues that influence the pattern of health-seeking behavior. Strategies suitable for LMICs may include focusing on inexpensive screening tools, task-shifting care to nonspecialist providers, and integrating screening services with established public health initiatives targeting higher-profile health issues.

### 6.2. The Role of Insurance Reimbursement in Lowering the Economic Barrier to OSA Treatment

There is evidence from the outside that broad insurance coverage for diagnosing and treating OSA is economically sound. Considering the comprehensive health system coverage, in the long term, such complications can be well avoided, and costs can be reduced by 34% [75]. Some value-based insurance designs reduce the copay for CPAP therapy and are associated with a 41% increase in adherence and decreased healthcare utilization [76]. Fresh funding models, such as equipment rental schemes and sliding-scale payments, have helped extend access to treatment. Such interventions have been found to increase the treatment initiation rate of low-income individuals by 28% [77].

### 6.3. Interventions at the Workplace to Reduce Losses in Productivity and Costs

Economic benefits have been demonstrated with workplace screening and management programs. Adopters of a comprehensive OSA program reduce costs by an average of USD 3400 per employee per year due to absenteeism and productivity improvement [29]. Some industries have reported a 3.5:1 benefit-to-cost ratio from two years of implementing sectoral or segmented workplace interventions [78]. Remote-led and support services for the workplace are effective as a technique for routine maintenance. Worldwide research indicates a 39% reduction in workplace accidents and a 27% reduction in disability claims for organizations that offer these programs [79].

### 6.4. Contribution of Public Health Awareness Campaigns to Reducing the Burden

More structured public health initiatives have been effectively introduced to increase OSA and treatment-seeking awareness. Interventions using more than one mode of communication have resulted in awareness of up to 56%, and a 33% reduction in time to diagnose [80]. Modeling has shown the tremendous cost-effectiveness of social media interventions compared to other outreach forms, with targeted campaigns garnering 2.8-fold engagement at one-third of the cost of conventional outreach [81]. The positive experience of community-based education programs is demonstrated by a 47% rise in treatment starts involving minority communities [82]. Economic modeling suggests that broad-based public health campaigns combined with increased access to treatment could reduce the economic burden of OSA on society by 23–31% over 5 years [10,83]. Incorporation of OSA recognition and management in standard care of chronic diseases can be mutually beneficial, bettering the outcomes of both diseases with a lower total cost for healthcare [84]. Research indicates that we need coordinated policy approaches that integrate healthcare systems, employers, and public health organizations. Cost-effectiveness analyses are, therefore, supportive of investment in broad-based programs, such that healthcare resource expenditure could be reduced by USD 6.3 billion per annum in the US alone [84]. Cross-national comparison studies have also suggested that countries with consistent and international policies may be able to effect a 50% greater reduction in OSA-related economic burden than countries with fragmented policies [85]. Recent findings advocate for tailored approaches tailored to each section of the population, highlighting superior outcomes and cost-effectiveness by applying more personalized intervention [86]. While they are implementing these recommendations at home, which would be ensured only by continued commitment to them and their resource allocation, the economic positive return on investment of these measures has been reported in a range of systems and economic settings [87].

## 7. Future Directions in OSA Research and Global Healthcare Strategies

From the perspective of our updated analysis of the global socioeconomic burden of OSA, we pinpoint the following three urgent research priorities:

- AI-based diagnostics and home-based screening programs in implementation science: There is a need for studies that move beyond efficacy in laboratory settings or under monitoring to effectiveness of these technologies in real life, especially in the underserved and in low-resource settings.
- Development and assessment of models of integrated care: How management of OSA can be integrated effectively into extant chronic disease programs, cardiovascular disease, and diabetes to improve outcomes and resource use.
- Full cost and benefit of innovative intervention: Long-term cost-effectiveness analysis of new pharmacologic treatments and minimally invasive surgical techniques is still needed to support the rational distribution of resources.

### 7.1. Research Needs in Low-Cost OSA Diagnostics and Treatments

While emerging technologies show promise for OSA diagnostics and treatment, significant research gaps must be addressed to establish their effectiveness and appropriate implementation. A newer, innovative wearable comprising high-order sensors and machine learning reported 89% diagnostic accuracy compared with the standard polysomnography in a restricted sample of clinicians, and developers state their potential as reductions in costs of 75% [88]. Nevertheless, these early promising findings warrant further investigation in larger, heterogeneous populations before such striking cost savings can be realized. This disruption holds the most promise in resource-constrained and in-home monitoring [89]. The enthusiasm about AI and telemedicine in OSA management must be balanced with sober reflection on the realities of implementation. The algorithms that characterize AI diagnostic tools are primarily fed with information from wealthy Western populations, potentially embedding biases that might decrease accuracy for those whose data is underrepresented. Telemedicine techniques, though seemingly widening reach, can widen disparities if implemented without regard to digital literacy, internet access, and cultural acceptance of different populations. Costs of these technologies rarely consider the significant investment in infrastructure necessary for equitable implementation.

The excitement about AI and telemedicine in the context of OSA management must be tempered by a sober consideration of the realities of adoption. The algorithms informing AI diagnostic tools are populated primarily with data from affluent Western populations, thus potentially introducing bias that may detract from accuracy for populations whose data is not well-representative. Telemedicine strategies, although they may seem to extend their reach, may instead create wider disparities if adopted without consideration of differing digital literacy, internet access, and cultural acceptance of different populations. Costs for these technologies rarely factor in significant capital investment in infrastructure to allow equitable rollout.

Innovative therapeutic options, including drug therapies aimed at specific OSA subtypes, may reduce the need for traditional CPAP treatment. Recent phase II clinical studies reported that new drug candidates demonstrated beneficial effects in specific patient subsets and potential for cost savings (estimated at 40–60% compared to life-long CPAP use) [90]. Advances in manufacturing, such as 3D printing, have made it easy to make an oral appliance for an individual patient, thus achieving a saving of 65% in cost without compromising therapeutic gains [91]. Real-time tracking devices carry out real-time monitoring and correction due to the integration of smart materials and biosensors, which can increase the treatment effectiveness and decrease the expense, increasing compliance [92].

### 7.2. Research Agenda for Telemedicine and AI-Based Solutions

To fulfill the potential of telemedicine and AI-based platforms in OSA care, research should address several critical questions. AI-augmented screening algorithms have demonstrated encouraging results in small research trials, with some studies claiming to distinguish high from low-risk subjects with an accuracy of up to 92% and reducing unnecessary testing by 43% [93]. These findings are derived from controlled settings and in select populations and may not be directly applicable to more heterogeneous real-world settings. ML models and algorithms have been created in the context of a remote monitoring system to predict CPAP adherence. Initial studies indicate the possibility of adherence gains of approximately 35% with provider burden reductions [94]; however, this work primarily derives from small studies with highly motivated subjects and may not imply results at larger scales. Preliminary assessments of the cloud-based ASI have indicated that approximately USD 450 per patient per year in savings could be realized by avoiding the manual scoring of records [95,96]. The implementation of natural language processing in electronic health records has also increased by 28% the efficiency of risk stratification and treatment planning. The potential use of virtual care solutions to address healthcare inequalities is evident with a 52% increase in access to specialized care, particularly in the underserved population [97]. Cost-effectiveness analyses suggest an expected annual product saving of USD 2800/patient related to lower travel and wait times [98].

Although promising in terms of addressing diagnostic delay and facilitating better access to care for OSA, AI and telemedicine modalities should be implemented in clinical practice from an ethical and practical point of view. Privacy is a real issue, and even more so with cloud-based systems and wearables recording very sensitive biometric data. The commitment to worldwide standards such as GDPR and HIPAA is important, especially if these tools are to be deployed in multiple countries. Also, many AI algorithms are developed using data from rich, largely Caucasian communities; so, fears of biased algorithms and poor performance for underrepresented groups are justified. Without validating, the risk of replicating the existing health inequities is substantial. Telemedicine platforms would also depend on stable internet access and digital health literacy, which could be lacking in some rural or low-income communities and potentially increase the digital divide. As such, future development must include transparent reporting, external validation in diverse populations, and ethical governance structures that promote fairness, equity, and inclusivity in the provision of OSA care.

Though promising, obstacles need to be overcome for successful implementation of telemedicine in these parts of the world. Access to the internet has also been a significant challenge, particularly in low-income or rural locations where broadband is not accessible or reliable. Even when digital infrastructure is improving in places, telemedicine platforms may rely on bandwidth and device capabilities that are beyond local access. Also, telehealth uptake may be impaired by limited digital literacy amongst patients and healthcare workers. Older and low-educated patients may have trouble learning to operate app-based interfaces or installing devices, and HCWs may not be trained in the utilization of remote monitoring technology. Confidence in these virtual systems, especially those that track health-related data, is also an issue that could affect patient adoption. However, the short term of many of the telemedicine pilots may also imply that these did not have sustainable funding or integration into national health systems. Without conducive financing and enabling policy environment, as well as the capacity building and empowerment at the community level, these innovations may still have the potential to widen the gap in rural areas rather than bridging it. Thus, the feasible telemedicine deployment should be multi-level and take into consideration technical, educational, cultural, and systemic challenges.

While it looks promising, challenges must be addressed before telemedicine can be effectively implemented in these regions of the world. Access to the internet has also been an important challenge, particularly in low-income or rural places where there is little or no broadband access or reliability. Even when digital infrastructure is improving in communities, telemedicine platforms may rely on bandwidth and device access that are not available to local populations. Additionally, telehealth uptake may also be limited by low levels of digital literacy among patients and HCWs. Older patients or patients with limited education may face difficulties learning to use app-based platforms and installing devices, and HCWs may not have any training in remote monitoring technology. Additionally, confidence in these virtual systems, particularly those that monitor health-related data, may contribute to low patient acceptance. However, the fact that many of these telemedicine pilots were ‘one–off’ or short-term initiatives may indicate that they did not have sustainable funding or were not linked to national health systems. Even with a sustainable funding mechanism, without an enabling policy environment, community-level capacity building, and empowerment, these innovations may not only be misused, but may also widen disparities in rural areas as opposed to bridging them.

Despite its potential, challenges must be adequately confronted to make telemedicine widely available in these regions. Access to the internet has also been a substantial obstacle, especially for low-income or rural areas where broadband is unavailable or unreliable. Even in places where digital infrastructure is getting better, telemedicine platforms may depend on bandwidth and device capability beyond local reach. Furthermore, low digital literacy in both patients and healthcare workers may hamper the adoption of telehealth. Older and low-educated adults may have difficulty with app-based interfaces or device installation, and HCWs may lack the training to effectively use remote monitoring technology. Trust in such digital systems, particularly ones dealing with sensitive health information, is also a concern that could further impact patient engagement. There is also the sustainability aspect. The short-term nature of many telemedicine pilots may suggest, however, a lack of sustainable funding or of integration with national health systems. In the absence of supportive reimbursement structures and regulation, and capacity building and empowerment at the community level, these solutions could run the risk of exacerbating rather than addressing the access gap in rural areas. Therefore, a viable telemedicine implementation should be multilevel and account for technical, educational, cultural, and systemic limitations.

### 7.3. Priorities for International Research Collaboration

To address the global burden of OSA effectively, international research collaborations should focus on these key priorities [47]. Global Sleep Health Initiative, a venture by establishes uniform diagnostic criteria and treatment guidelines in 45 countries, improves care, delivery, and cost [99]. These cooperative research programs have facilitated the acceleration of population-specific improvements and have been the most successful for individuals of Asian and African descent [100]. This sharing of data moved beyond single healthcare systems and strengthened predictive models and outcome analysis. By examining the International Sleep Research Database, cost-effective treatment strategies applicable in different healthcare settings have been identified that could potentially lead to global annual cost-savings of USD 12.5 billion [101]. Organized, multicenter analyses and investigations of genetic and environmental contributions to OSA have also led to more specific therapeutic adjuncts. Indeed, the World Sleep Health Consortium has identified new biomarkers that could, based on improved patient stratification, cut the cost of diagnosis by 55% [102]. Global harmonized approaches towards smart and safe OSA management are reflected in more recent policy frameworks. Global standardization of care pathways may present a model that has the potential to significantly impact costs to health; according to an economic model, if such standardization were possible, global 5-year health costs of treating OSA could be reduced by 28% [103]. Cross-border resources and expertise sharing were particularly shown to help develop healthcare systems [104]. The future of OSA treatment will likely be shaped by technological innovations, standardized protocols, and international collaborative research networks. That technology adoption may decrease the global economic burden of OSA by 35–40% in 2030 [104]. Nevertheless, such models often assume perfect adoption, effectiveness, and access to the technology that may not reflect the complexity of healthcare systems and inequalities around access to technology [104]. This challenge has been met by continued investment in R&D and the cooperation and sharing of knowledge between nations [10].

International research collaboratives can provide valuable opportunities for knowledge exchange, but critically reflecting on such collaboratives underscores the enduring power differentials that exist that may inhibit meaningful cooperative exchange through the research process. They often emphasize research questions that travel well in high-income settings but do not address important contextual challenges in low-resourced situations. Furthermore, a clinical standardization of diagnostic criteria and treatment guidelines across disparate healthcare systems runs the risk of exporting Western medical practice in ways that do not fit available, local resources, cultural framings of reality, and health priorities. When confronting these issues, meaningful equity in global partnership truly requires a fundamental rethinking of how research agendas are determined and how and where benefits are directed.

While international research collaboratives offer valuable opportunities for knowledge exchange, critical evaluation reveals persistent power imbalances that may undermine their effectiveness. These collaborations often prioritize research questions relevant to high-income settings while failing to address context-specific challenges in low-resource regions. Additionally, the standardization of diagnostic criteria and treatment guidelines across diverse healthcare systems risks imposing Western medical paradigms that may not align with local resources, cultural perspectives, or health priorities. Truly equitable global approaches require fundamental reconsideration of how research agendas are established and how benefits are distributed.

## 8. Conclusions

This extensive review supports that the economics of OSA have been proven to be a significant burden worldwide, not only due to direct healthcare costs but also due to indirect costs due to productivity and accident loss. Our report emphasizes the marked variability in the burden of OSA in diagnosis and access to treatment in regions and according to socioeconomic status. The evidence allows several important observations, including the following: (1) early intervention and good care are a good buy in a range of healthcare models; (2) policy solutions that couple OSA screening with existing chronic disease actions stand out; and (3) implementation of technology-enhanced solutions must be handled carefully to avoid widening disparities. Although new technologies have the potential to increase access, their efficacy and cost-effectiveness in heterogeneous real-world contexts have yet to be empirically determined. Although there is a large body of evidence of OSA’s effects on the socioeconomic aspects, the burden is largely unknown among different populations and healthcare infrastructure. The dominance of high-income country data, variability in economic evaluation methods, and lack of focus on region-specific barriers to diagnosis and care restrict the setting of internationally applicable strategies. To overcome these restricted evidence gaps of high quality, future research efforts should aim to be more generalizable, to provide more consistent economic analyses, and to better address issues of uptake in resource-strained settings. Only with this questioning approach will we be able to design fair strategies to tackle the enormous socioeconomic burden of OSA at a global level.

Although a large body of evidence is available, OSA has a substantial socioeconomic effect; however, we do not fully understand the disease burden related to OSA in different patient groups and healthcare systems. There is an overwhelming focus on high-income countries and a mismatch regarding methods of economic valuation, but no consideration of issues that affect access to diagnosis and management in that region, to generate international strategies with global applicability. To address the knowledge gaps, there needs to be a wider range of geographic locations, greater consistency in economic analyses, and a better understanding of the difficulties involved in addressing issues related to implementation in resource-poor settings. Only then, with a broader context, can we hope to develop fair strategies to manage the massive socioeconomic burden of OSA globally.

We hope this review will encourage healthcare professionals and decision makers to make prudent early OSA treatment decisions and to act as a catalyst for further work in this area, as intervention not only leads to improved clinical outcomes but also to significant economic savings. It serves as a useful guide to develop evidence-based public health approaches to take OSA up in the national and international health agenda.

Despite the availability of extensive evidence, OSA has significant socioeconomic impacts; the disease burden due to OSA is incompletely understood in different patient groups and healthcare systems. The high-income country data dominance and inconsistent use of methods of economic valuation, while not accounting for region-specific barriers to diagnosis and management, limit the development of strategies with global relevance. Toward addressing these evidence gaps, a broader range of geographic settings, more consistent economic analyses, and a better understanding of issues related to implementation in resource-poor settings are needed to guide future research efforts. With only this broader perspective can we develop fair strategies to address the huge socioeconomic burden of OSA worldwide.

## Data Availability

No new data were created for this article.

## Abbreviations

The following abbreviations are used in this manuscript:

OSA	Obstructive Sleep Apnea
OSAS	Obstructive Sleep Apnea Syndrome
CPAP	Continuous Positive Airway Pressure
MAD	Mandibular Advancement Devices
UPPP	Uvulopalatopharyngoplasty
ICER	Incremental Cost-Effectiveness Ratio
QALY	Quality-Adjusted Life Year
BMI	Body Mass Index
GDP	Gross Domestic Product
AI	Artificial Intelligence
USA	United States of America
RA	Rheumatoid Arthritis
